# Marine algal flora of Formigas Islets, Azores

**DOI:** 10.3897/BDJ.8.e57510

**Published:** 2020-10-02

**Authors:** Ana I. Azevedo Neto, Afonso C. L. Prestes, José M. N. Azevedo, Roberto Resendes, Nuno Vaz Álvaro, Raul M. A. Neto, Ignacio Moreu

**Affiliations:** 1 cE3c - Centre for Ecology, Evolution and Environmental Changes/Azorean Biodiversity Group & Faculdade de Ciências e Tecnologia, Departamento de Biologia, Universidade dos Açores, 9500-321 Ponta Delgada, São Miguel, Açores, Portugal cE3c - Centre for Ecology, Evolution and Environmental Changes/Azorean Biodiversity Group & Faculdade de Ciências e Tecnologia, Departamento de Biologia, Universidade dos Açores 9500-321 Ponta Delgada, São Miguel, Açores Portugal; 2 Universidade dos Açores, Faculdade de Ciências e Tecnologia, Departamento de Biologia, 9500-321 Ponta Delgada, São Miguel, Açores, Portugal Universidade dos Açores, Faculdade de Ciências e Tecnologia, Departamento de Biologia 9500-321 Ponta Delgada, São Miguel, Açores Portugal; 3 Universidade dos Açores, Faculdade de Ciências Agrárias, CCMMG (Centro do Clima Meteorologia e Mudanças Globais), IITA-A (Instituto de Investigação e Tecnologias Agrárias e do Ambiente), 9700-042 Angra dp Heroísmo, Terceira, Portugal Universidade dos Açores, Faculdade de Ciências Agrárias, CCMMG (Centro do Clima Meteorologia e Mudanças Globais), IITA-A (Instituto de Investigação e Tecnologias Agrárias e do Ambiente) 9700-042 Angra dp Heroísmo, Terceira Portugal; 4 N/A, Odivelas, Portugal N/A Odivelas Portugal

**Keywords:** Macroalgae, new records, Azores, Formigas Islets, endemism, native, introduced, uncertain, occurrence data.

## Abstract

**Background:**

The oldest reference to marine life in Formigas Islets (oriental group of the Azores archipelago) goes back to the 16th century. Nevertheless, their macroalgal flora is poorly known, the published information mainly resulting from occasional collections of sporadic visitors. To overcome this and contribute to the knowledge of Azorean macroalgal flora at both local and regional scales, a thorough investigation was conducted in 1990 and 1991 under two expeditions promoted by the Marine Biology Research Group of the Department of Biology, University of the Azores. Collections and presence data recordings were undertaken at the littoral and sublittoral levels down to approximately 40 m, in an area of approximately 0.04 km^2^. This paper lists the taxonomic records and provides information regarding each species’ ecology and occurrence on the Islets’ littoral.

**New information:**

A total of 320 specimens are registered (including taxa identified only at generic level) belonging to 90 taxa of macroalgae, from which 70 were diagnosed at species level. The confirmed species comprise 39 Rhodophyta, 12 Chlorophyta and 19 Ochrophyta (Phaeophyceae), distributed in 22 orders (13 Rhodophyta, 3 Chlorophyta and 6 Ochrophyta) and 37 families (24 Rhodophyta, 6 Chlorophyta and 7 Ochrophyta). Sixty-one species represent new records for the Islets, from which *Botryocladia macaronesica* Afonso-Carrillo, Sobrino, Tittley & Neto and *Laurencia viridis* Gil-Rodriguez & Haroun are Macaronesian endemisms. Most species are native to the Azores, but six have an uncertain origin and four are introduced (the Rhodophyta *Asparagopsis armata* Harvey; *Laurencia dendroidea* J.Agardh; *Neoizziella divaricata* (C.K.Tseng) S.-M.Lin, S.-Y.Yang & Huisman and the Ochrophyta *Hydroclathrus tilesii* (Endlicher) Santiañez & M.J.Wynne).

## Introduction

The Formigas Islets are located about 31 km NE of Santa Maria Island and 55 km SE of São Miguel Island (oriental group of Azorean archipelago, approximately 37°16′35″N, 24°46′54″W). They are arranged in a N-S direction, over a total length of about 165 m and a width of 80 m. Together with the submersed bank of Dollabarat, they form the Nature Reserve of Formigas Bank (DLR n° 11/88/ A).

The oldest reference to life in Formigas Islets consists of descriptions of its marine fauna in the 16th century manuscript "*Saudades da terra*", written by the naturalist clergyman Gaspar Frutuoso. Subsequently, these Islets were occasionally studied in sporadic visits by researchers, the first reference to the marine macroalgae being that of Piccone (1889). After that, several expeditions were made in order to study of the fauna and flora of the Islets, which resulted in a few publications (see revision in Azevedo et al. 1991). An important finding was the first Azorean record of the brown alga *Laminaria ochroleuca* Bachelot de la Pylaie (made by Ardré et al. 1973). Despite these efforts, the algal flora of these Islets remained poorly known until the nineties, when a thorough investigation conducted by the Marine Biology Research Group of the Department of Biology, University of the Azores, took place. This research group went to the Islets in 1990 and 1991 and undertook collections and presence data recordings at the littoral and sublittoral levels down to about 40 m, over an area of approximately 0.04 km^2^.

## General description

### Purpose

This paper, aimed at contributing to a better understanding of the seaweed flora of the Azores archipelago, lists the macroalgae recorded on surveys undertaken on the Formigas Islets (Azores, eastern group) and presents general information for each taxon’s occurrence on the Islets’ littoral, thus addressing several biodiversity shortfalls (see Hortal et al. 2015), namely the need to catalogue the Azorean macroalgae (Linnean shortfall) and improve the current information on their local and regional geographic distribution (Wallacean shortfall), as well as on species abundances and dynamics in space (Prestonian shortfall). It is intended as a resource for academics, students, government, private organisations and the general public and also as a practical basis for biological studies, such as systematics, diversity and conservation, biological monitoring, climate change and ecology.

## Project description

### Title

Marine algal (seaweed) flora of Formigas Islets, Azores

### Personnel

Sampling took place in the summers of 1990 and 1991 under the coordination of Ana I. Neto. Main collectors were Ana I. Neto, Bruno Brum, Carlos Rodrigues, Heather Baldwin, João Brum, José M. N. Azevedo, José Pedro Viegas and Luís Resendes.

Ana I. Neto and Heather Baldwin were responsible for the species identification.

Voucher specimen management was mainly undertaken by Afonso Prestes, Ana I. Neto, Eunice Nogueira, Natália Cabral and Roberto Resendes.

### Study area description

**Figure 1.**
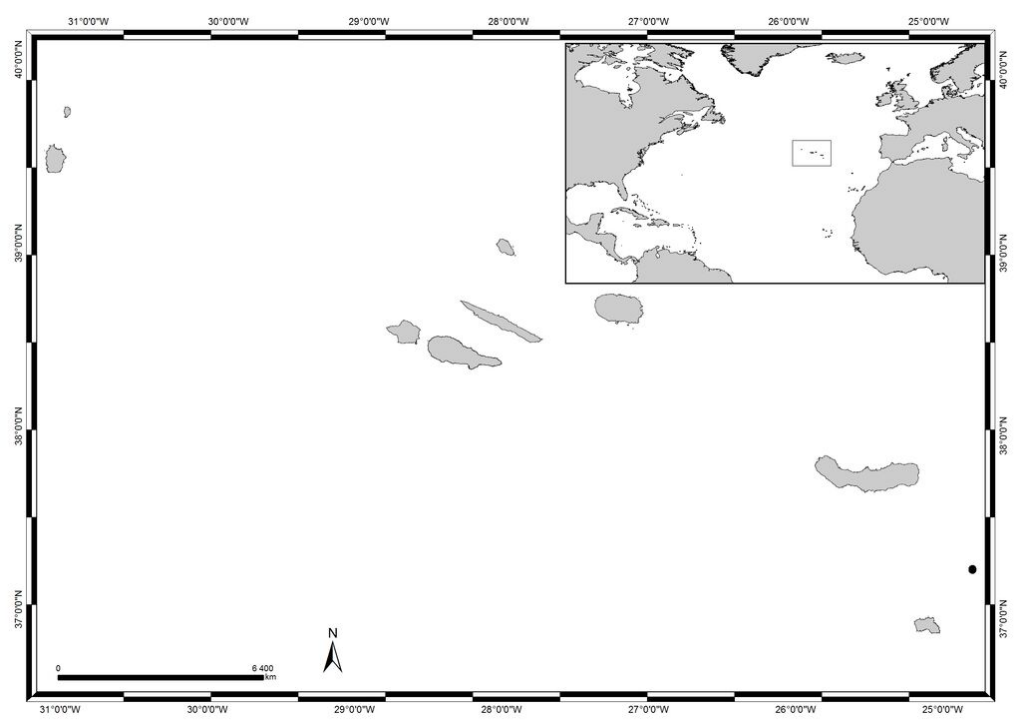
The Azores, its location in the Atlantic and Formigas Islets highlighted in black (by Nuno V. Álvaro).

Located in the eastern group of the Azores archipelago, the Formigas Islets (37°16′35″N, 24°46′54″W, Fig. 1) are approximately 32 km NE of Santa Maria Island and 55 km SE of São Miguel Island, thus being the most isolated Islets of the Azores. Consequently, they are relatively protected from human action and function as a breeding and nursing ground for many marine species occurring in the Azorean waters (Costa et al. 1994). The Islets, together with the submersed bank of Dollabarat, form the Formigas Bank, located between the parallels 37°14'N and 37°17'N and the meridians 24°43'W and 24°47'W, occupying an area about 11 km long and 5 km wide (Azevedo et al. 1991) and designated as a Nature Reserve in 1988 (DLR n° 11/88/ A).

**Figure 2.**
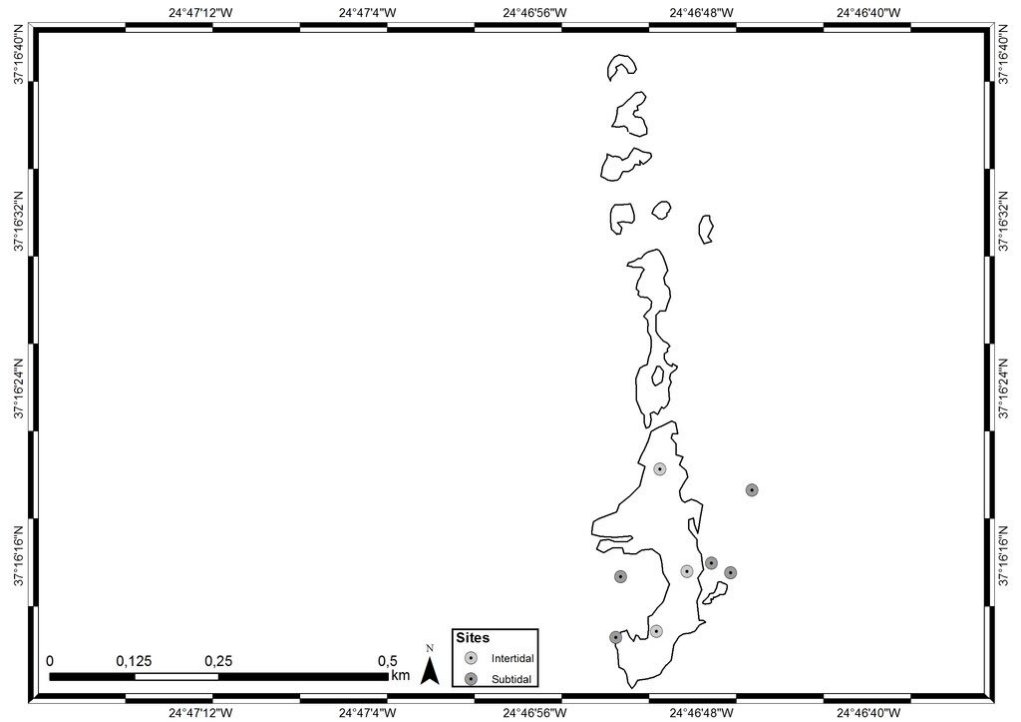
Formigas Islets with indication of the sampling locations (by Nuno V. Álvaro).

The Formigas Islets are located in the NW part of the bank. With a N-S arrangement, they have a total length of about 165 m and width of 80 m, with an area of compact rocks in the southern part and one of large blocks in the North (Fig. 2). The highest block (Formigão) is 11 m high. In the South region, there is a lighthouse, from which there are two small anchorages (Azevedo et al. 1991).

As in the remaining archipelago, the climate is temperate oceanic with persistent winds, regular and abundant rainfall and high levels of relative humidity, mainly during winter and autumn (Morton et al. 1998). The tidal range is small (< 2 m, see Hidrográfico 1981) and the Islets are surrounded by deep waters. Intertidal space is limited and permanently subjected to the action of the waves, which does not allow the establishment of terrestrial fauna and flora (Costa et al. 1994), but the marine biota is rich. Most seaweeds and invertebrates that characterise the exposed Azorean coasts can be found there; the great clarity of the waters allows the algal communities to extend to great depths. This profusion of algae provides shelter and food for many animals and supports a complex food web (Costa et al. 1994).

**Figure 3.**
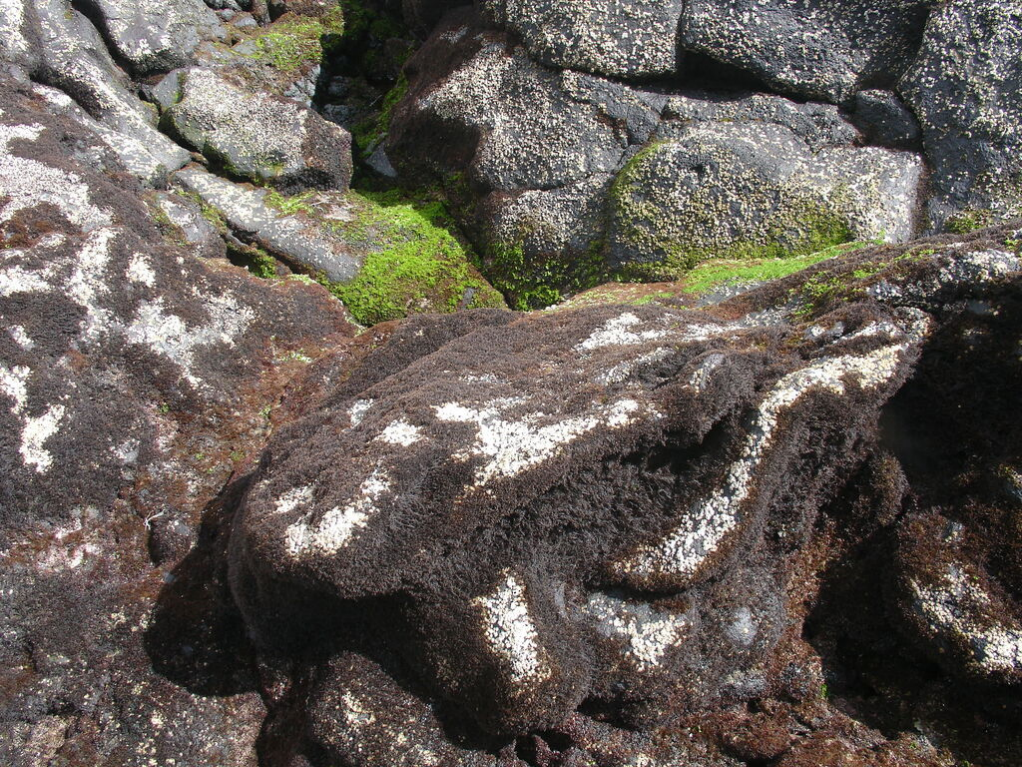
Mid-shore intertidal level showing gastropods, chthamalid barnacles and algal turf (by the Island Aquatic Ecology Subgroup of cE3c-ABG).

**Figure 4.**
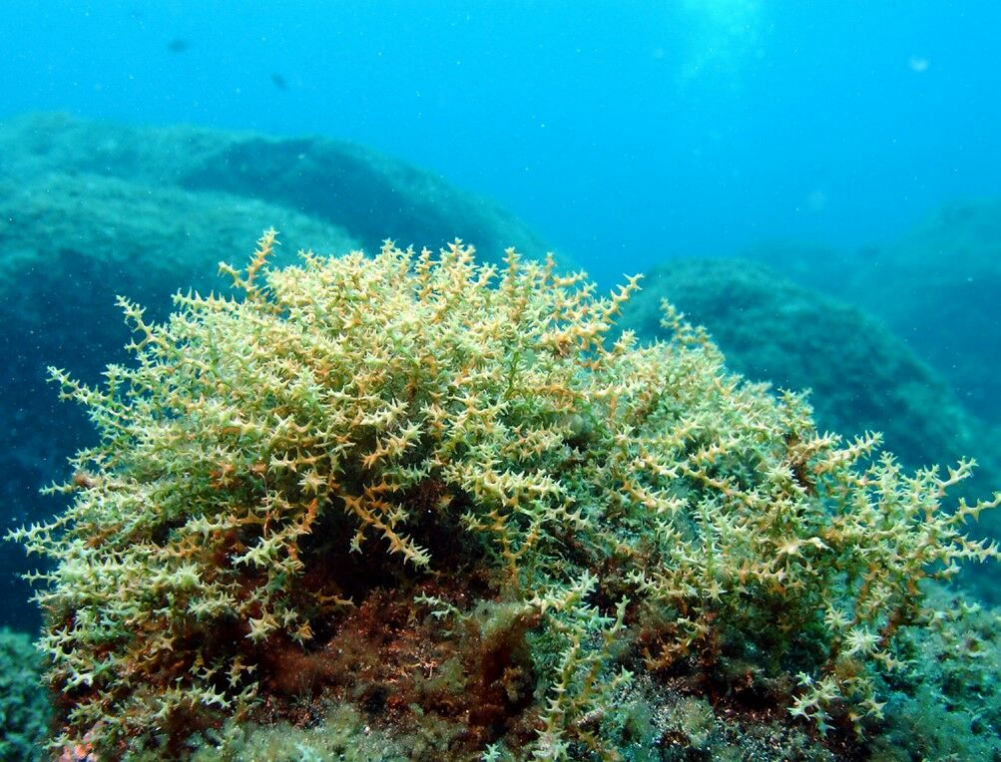
*Treptacantha abies-marina* in an intertidal channel (by the Island Aquatic Ecology Subgroup of cE3c-ABG).

**Figure 5.**
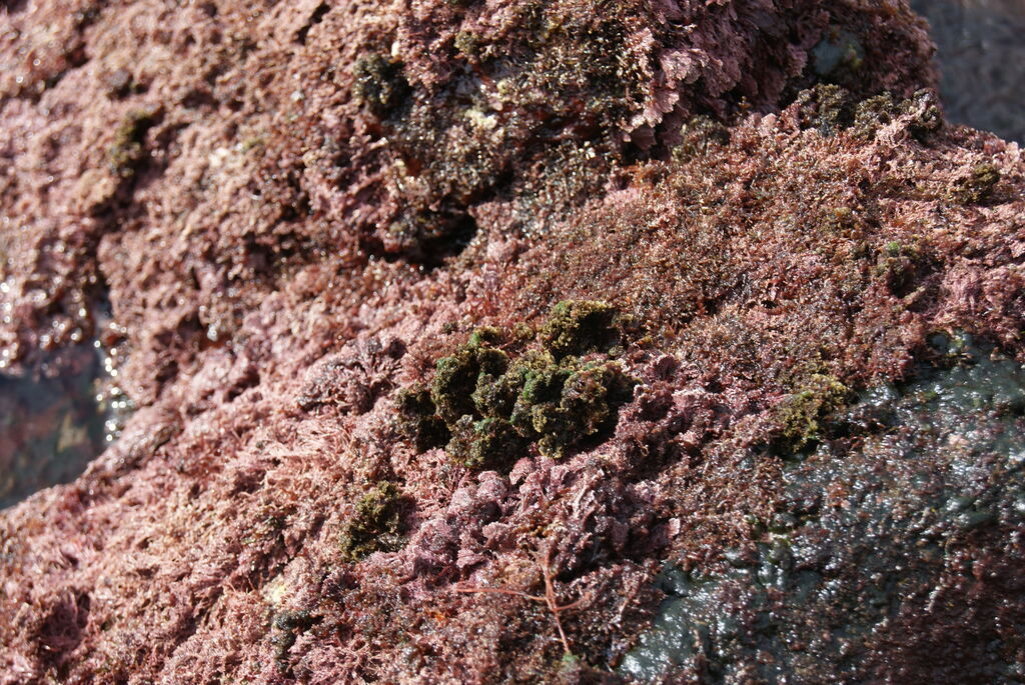
Low intertidal showing multi-specific algal turf and *Elisolandia elongata* (by the Island Aquatic Ecology Subgroup of cE3c-ABG).

**Figure 6.**
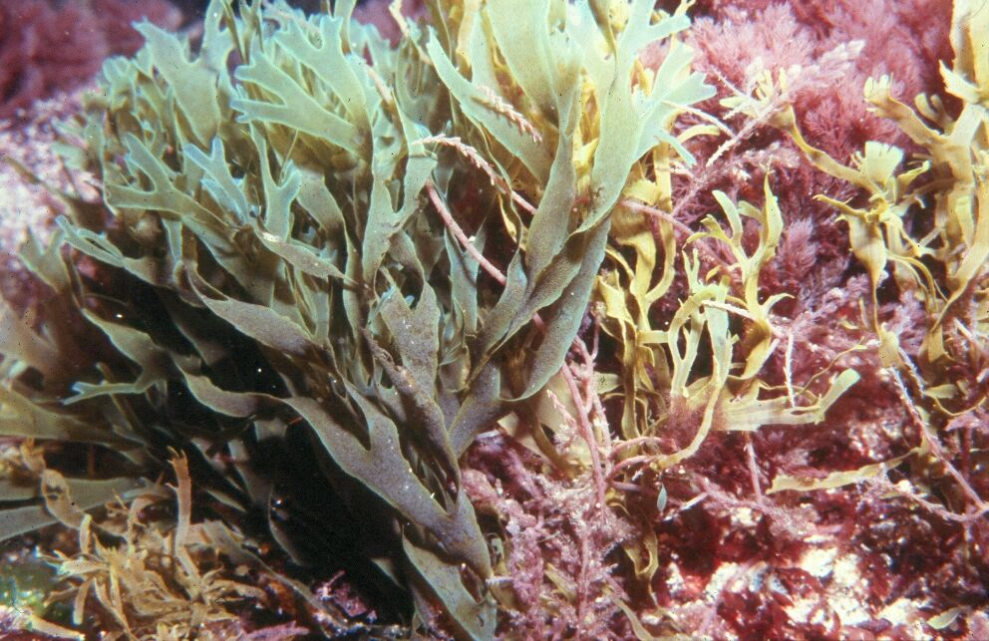
Frondose macrophytes (*Dictyota* spp. and *Plocamium cartilagineum*) at the subtidal level (by the Island Aquatic Ecology Subgroup of cE3c-ABG).

The intertidal zone is narrow and mostly dominated by animals (e.g. gastropods, chthamalid barnacles and decapods) and algal turfs (mostly composed by various species of *Ceramium* spp. and *Gymnogongrus* spp.) (Fig. 3), that are typically found in most of the Islands. The many channels that cross the rocky platforms are dominated by luxuriant forms of the brown algae *Cystoseira* spp., *Treptacantha abies-marina* (Fig. 4) and *Sargassum* spp. At the low intertidal, the algal turfs give rise to erect forms of algae, for example, *Elisolandia elongata* (Fig. 5). Subtidally, the rocky walls and rocky platforms are covered with erect, corticated macrophytes, for example, *Dictyota* spp. and *Plocamium cartilagineum* (Fig. 6). At and below 40 m depth, the leathery brown algae *Laminaria ochroleuca* can form some monospecific patches (Neto, pers. observ.).

### Design description

The macroalgae, referred to in this paper, were collected during field studies at littoral and sublittoral levels down to approximately 40 m on the Formigas Islets, over an area of 0.04 km^2^ (Table 1, Fig. 2). Intertidal collections were undertaken at low tide by walking over the shore. Subtidal collections were undertaken by scuba diving. Each sampling location was visited several times. On each occasion, a careful survey was made to allow a good coverage of the area. Whenever an unknown species was found, it was collected, given an individual registration number and vouchers deposited at the AZB Herbarium Ruy Telles Palhinha, at the Faculty of Sciences and Technology of the University of the Azores.

### Funding

This study was mainly financed by the following projects/scientific expeditions:

- Expedition SANTA MARIA and FORMIGAS/90, Departamento de Biologia da Universidade dos Açores, Ilha de Santa Maria e Ilhéus das Formigas, Açores, June 1990;
- Expedition FORMIGAS/91, Secção de Biologia Marinha do Departamento de Biologia da Universidade dos Açores, Ilhéus das Formigas, July 1991;
- Project “ACORES-01-0145-FEDER-000072 - AZORES BIOPORTAL – PORBIOTA. Operational Programme Azores 2020 (85% ERDF and 15% regional funds);
- Portuguese National Funds, through FCT – Fundação para a Ciência e a Tecnologia, within the projects UID/BIA/00329/2019 and UID/BIA/00329/2020-2023;
- CIRN/DB/UAc (Research Centre for Natural Resources, Universidade dos Açores, Departamento de Biologia).

## Sampling methods

### Study extent

Collections were made at littoral and sublittoral levels down to about 40 m around Formigas Islets over a total area of approximately 0.04 km^2^ (Fig. 2).

### Sampling description

**Figure 7.**
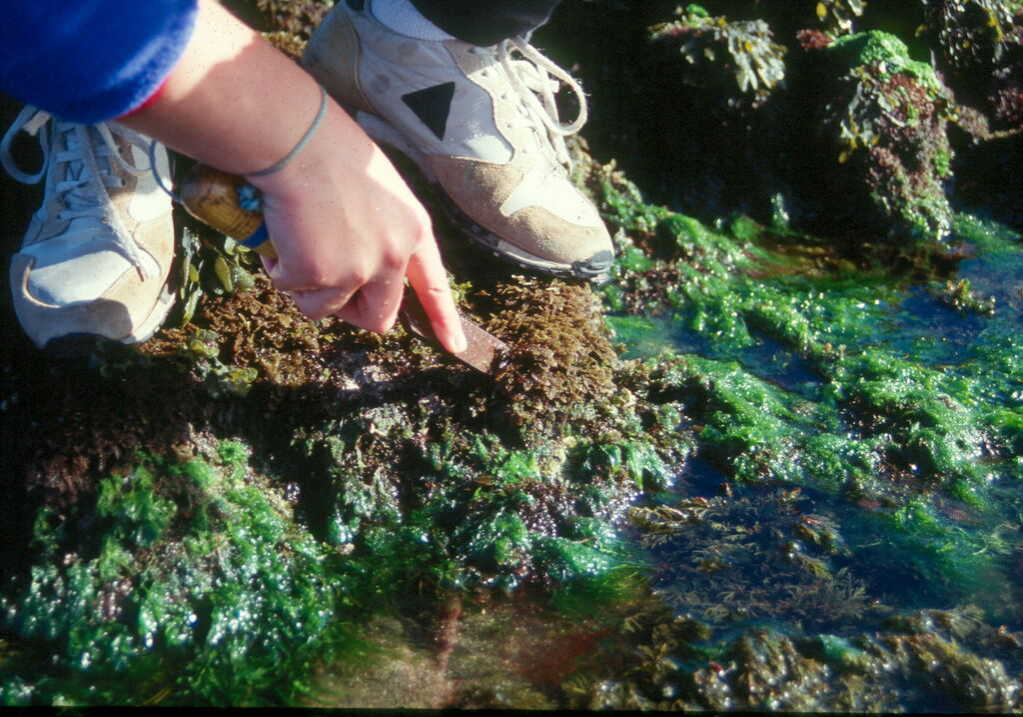
Collecting macroalgae at the rocky intertidal (by the Island Aquatic Ecology Subgroup of cE3c-ABG).

Intertidal collections were made at low tide by walking over the shore. Subtidal collections were made by scuba diving. In each sampling location, one or two specimens of all different species found were scraped into labelled bags (Fig. 7). Complementary data, such as shore level (high, mid, low), orientation and type of substrate (bedrock, boulders, mixed), habitat (tide pool, open rock, gully, crevice) were also recorded.

### Quality control

Each sampled taxon was investigated by trained taxonomists with the help of keys and floras. This involved morphological and anatomical examination by eye or under the dissecting and compound microscopes of an entire specimen or slide preparation. In difficult cases, specimens were sent to experts for identification.

### Step description

In the laboratory, the specimens were sorted and studied following standard procedures used in macroalgae identification.

Species identification was based on morphological and anatomical characters and reproductive structures. For small and simple thalli, this required the observation of the entire thallus with the naked eye and/or using dissecting and compound microscopes. For larger and more complex algae, the investigation of thalli anatomy required histological work to obtain longitudinal and transverse sections needed for the observation of cells, reproductive structures and other diagnostic characters.

Due to the mixed nature of the Azorean macroalgal flora, relevant floras from the Atlantic and western Mediterranean were used for the species identification (e.g. Schmidt 1931, Taylor 1967, Taylor 1978, Levring 1974, Dixon and Irvine 1977, Lawson and John 1982, Irvine 1983, Gayral and Cosson 1986, Fletcher 1987, Afonso-Carrillo and Sansón 1989, Burrows 1991, Boudouresque et al. 1992, Cabioc'h et al. 1992, Maggs and Hommersand 1993, Irvine and Chamberlain 1994, Brodie et al. 2007, Lloréns et al. 2012, Rodríguez-Prieto et al. 2013).

For more critical and taxonomically-difficult taxa, specimens were taken to the herbarium of the Natural History Museum (London) for comparison with collections there.

A reference collection was made for all specimens collected by giving them a herbarium code number and depositing them at the AZB Herbarium Ruy Telles Palhinha, University of Azores. Depending on the species and on further research planned, different types of collections were made, namely (i) liquid collections using 5% buffered formaldehyde seawater and then replacing it by the fixing agent Kew (Bridsen and Forman 1999) and (ii) dried collections, either by pressing the algae (most species) following the method described by Gayral and Cosson (1986).

Nomenclatural and taxonomic status used here follow *Algaebase* (Guiry and Guiry 2020). The database was organised on FileMaker Pro.

## Geographic coverage

### Description

Formigas Islets, Azores, Macaronesia, Portugal (approximately 37°16′35″N, 24°46′54″W).

### Coordinates

37.269 and 37.276 Latitude; -24.783 and -24.778 Longitude.

## Taxonomic coverage

### Description

All macroalgae were identified to genus or species level. In total, 90 taxa were identified comprising 71 confirmed species, belonging to 22 orders and 37 families, distributed by the phyla Rhodophyta (13 orders and 24 families), Chlorophyta (3 orders and 6 families) and Ochrophyta (6 orders and 7 families) (see Tables 2, 3).

### Taxa included

**Table:** 

Rank	Scientific Name	Common Name
phylum	Rhodophyta	Red algae
phylum	Chlorophyta	Green algae
phylum	Ochrophyta	Brown algae

## Temporal coverage

**Data range:** 1990-7-07 – 1990-7-14; 1991-7-05 – 1990-7-12.

### Notes

The sampling was performed in the summers of 1990 and 1991.

## Collection data

### Collection name

AZB | Marine macroalgae collection of Formigas Islets - Expedition SANTA MARIA and FORMIGAS/90; AZB | Marine macroalgae collection of Formigas Islets - Expedition FORMIGAS/91.

### Collection identifier

de350d60-48c0-409c-a71f-0ae4df753fde; 2d4aad32-17f3-426e-92f1-3d8654fc781e.

### Parent collection identifier

AZB Herbarium Ruy Telles Palhinha, Faculty of Sciences and Technology of the University of the Azores; AZB Herbarium Ruy Telles Palhinha, Faculty of Sciences and Technology of the University of the Azores.

### Specimen preservation method

Air-dry, Dried and pressed; Liquid (Formalin; fixing agent Kew), Silica

### Curatorial unit

AZB Herbarium Ruy Telles Palhinha, Faculty of Sciences and Technology of the University of the Azores.

## Usage rights

### Use license

Creative Commons Public Domain Waiver (CC-Zero)

## Data resources

### Data package title

Marine algal (seaweed) flora of Formigas Islets, Azores

### Resource link

http://ipt.gbif.pt/ipt/resource?r=formigas_seaweed_flora

### Alternative identifiers

https://www.gbif.org/dataset/22c0c715-b2a8-4c01-9719-13f79be07fdc

### Number of data sets

1

### Data set 1.

#### Data set name

Marine algal (seaweed) flora of Formigas Islets, Azores

#### Data format

Darwin Core Archive

#### Number of columns

49

#### Download URL

http://ipt.gbif.pt/ipt/resource?r=formigas_seaweed_flora

#### Data format version

version 1.7

#### Description

This data paper presents data from macroalgae surveys developed in Formigas Islets in 1990 and 1991 (Neto et al. 2020). The dataset submitted to GBIF is structured as a sample event dataset, with two tables: event (as core) and occurrences. The data in this sampling event resource have been published as a Darwin Core Archive (DwCA), which is a standardised format for sharing biodiversity data as a set of one or more data tables. The core data table contains eight records (eventID). The extension data table has 320 occurrences. An extension record supplies extra information about a core record. The number of records in each extension data table is illustrated in the IPT link. This IPT archives the data and thus serves as the data repository. The data and resource metadata are available for downloading in the downloads section.

**Data set 1.** 

Column label	Column description
Table of Sampling Events	Table with sampling events data (beginning of table)
eventID	Identifier of the event, unique for the dataset
country	Country of the sampling site
countryCode	Code of the country where the event occurred
stateProvince	Name of the region
island	Name of the island
municipality	Name of the municipality
locality	Name of the locality
locationID	Identifier of the location
decimalLatitude	The geographic latitude of the sampling site
decimalLongitude	The geographic longitude of the sampling site
geodeticDatum	The spatial reference system upon which the geographic coordinates are based
coordinateUncertaintyInMetres	The horizontal distance (in metres) from the given decimalLatitude and decimalLongitude describing the smallest circle containing the whole of the Location
eventDate	Time interval when the event occurred
year	The year of the event
samplingProtocol	Sampling method used during an event
locationRemarks	Zonation level
minimumDepthInMetres	The minimum depth in metres where the specimen was found
maximumDepthInMetres	The maximum depth in metres where the specimen was found
eventRemarks	Notes about the event
Table of Species Occurrence	Table with species occurrence data (beginning of new table)
occurrenceID	Identifier of the record, coded as a global unique identifier
institutionID	The identifier for the institution having custody of the object or information referred to in the record
institutionCode	The acronym of the institution having custody of the object or information referred to in the record
collectionID	An identifier of the collection to which the record belongs
collectionCode	The name of the collection from which the record was derived
datasetName	The name identifying the dataset from which the record was derived
eventID	Identifier of the event, unique for the dataset
kingdom	Kingdom name
phylum	Phylum name
class	Class name
order	Order name
family	Family name
genus	Genus name
specificEpithet	The name of the first or species epithet of the scientificName
infraspecificEpithet	The name of the lowest or terminal infraspecific epithet of the scientificName, excluding any rank designation
acceptedNameUsage	The specimen accepted name, with authorship
previousIdentifications	Previous name of the specimen, with authorship
scientificName	The name without authorship applied on the first identification of the specimen
basisOfRecord	The specific nature of the data record
habitat	Description of the habitat where the specimen was found
recordedBy	Person(s) responsible for sampling
catalogNumber	Identifying code for a unique sample lot in a biological collection
identifiedBy	Person(s) responsible for taxa identification
type	The nature of the resource
preparations	The preservation method used for the specimen
establishmentMeans	The establishment status of the organism in the study region
occurrenceRemarks	New record status assignment
licence	Reference to the licence under which the record is published

## Additional information

**Figure 8.**
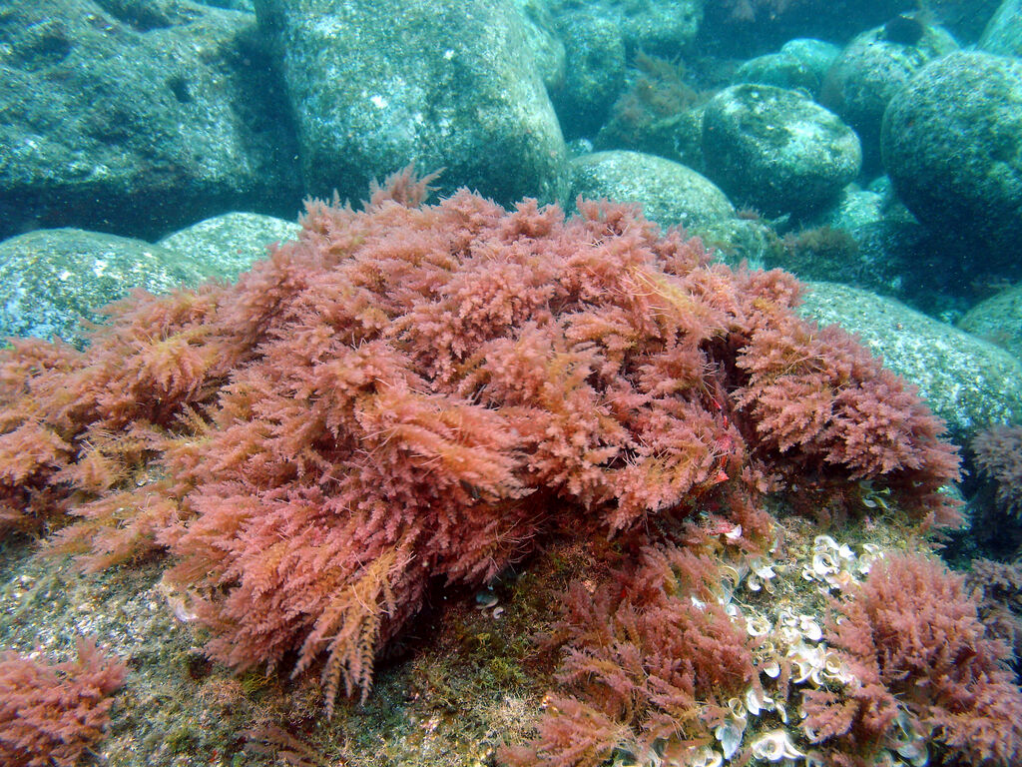
*Asparagopsis armata*, a new record for the Formigas Islets (by the Island Aquatic Ecology Subgroup of cE3c-ABG).

This paper is based on the 320 specimens of macroalgae collected from the Formigas Islets. Ninety taxa are listed (54 Rhodophyta, 14 Chlorophyta and 22 Ochrophyta) of which 70 are confirmed species and 20 taxa are identified only to genus level. The confirmed species (Tables 2, 3) include 39 Rhodophyta, 12 Chlorophyta and 19 Ochrophyta (Phaeophyceae) distributed by 22 orders (13 Rhodophyta, 3 Chlorophyta and 6 Ochrophyta) and 37 families (24 Rhodophyta, 6 Chlorophyta and 7 Ochrophyta). Sixty-one of the confirmed species are newly-recorded to the islets (37 Rhodophyta, 11 Chlorophyta and 13 Ochrophyta), as the invasive *Asparagopsis armata* (Fig. 8). Most species are native (58), including the Macaronesian endemisms *Botryocladia macaronesica* Afonso-Carrillo, Sobrino, Tittley & Neto and *Laurencia viridis* Gil-Rodríguez & Haroun. Four species represent introductions to the algal flora (the Rhodophyta *Asparagopsis armata* Harvey, *Laurencia dendroidea* J.Agardh, *Neoizziella divaricata* (C.K.Tseng) S.-M.Lin, S.-Y.Yang & Huisman and the Ochrophyta *Hydroclathrus tilesii* (Endlicher) Santiañez & M.J.Wynne) and six have an uncertain status.

Many species were only sporadically observed on the Islets, but seven were commonly found, namely: the Rhodophyta *Asparagopsis armata* Harvey, *Cryptopleura ramosa* (Hudson) L.Newton, *Plocamium cartilagineum* (Linnaeus) P.S.Dixon and *Pterocladiella capillacea* (S.G.Gmelin) Santelices & Hommersand; and the Ochrophyta *Dictyota bartayresiana* J.V.Lamouroux, *Treptacantha abies-marina* (S.G.Gmelin) Kützing and *Zonaria tournefortii* (J.V.Lamouroux) Montagne.

A mismatch regarding the GBIF backbone taxonomy of some of the macroalgae species names was identified as detailed in Suppl. material 1.

## Supplementary Material

CEEBDD2F-7083-5FF0-8D10-5913AF12A6F3 10.3897/BDJ.8.e57510.suppl1 Supplementary material 1 DP-FOR-id_14155_normalized-redz.csv Data type Macroalgae taxonomic mismatching Brief description GBIF does not have the more actualised nomenclature for some of the macroalgae species names. Therefore, the matching tools of its platform were applied to the species list, as required by Pensoft's data auditor, to identify the problematic taxonomic situations. The resulting file (DP-FOR-id_14155_normalized-redz.csv) is included here, since the names will not be immediately updated in the GBIF Taxonomic Backbone. A request was already sent to GBIF helpdesk to resolve this situation. File: oo_441364.csv https://binary.pensoft.net/file/441364 Ana I. Neto

## Figures and Tables

**Table 1.** Formigas Islets' sampling sites information.

Location N0	Location ID	Municipality	Locality	Latitude / Longitude	geodeticDatum	Littoral zone
1	FOR_FOR_PNI	Formigas	Ponta norte do ilhéu	37,272261, -24,780546	WGS294	Intertidal
3	FOR_FOR_AVI1	Formigas	À volta do ilhéu 1	37,270887, -24,779604	WGS84	Subtidal
4	FOR_FOR_AVI2	Formigas	À volta do ilhéu 2	37,271983, -24,779321	WGS84	Subtidal
2	FOR_FOR_AVI3	Formigas	À volta do ilhéu 3	37,27011, -24,780593	WGS84	Intertidal
5	FOR_FOR_II	Formigas	No intertidal do ilhéu	37,270904, -24,780187	WGS84	Intertidal
6	FOR_FOR_II	Formigas	No intertidal do ilhéu	37,271014, -24,779862	WGS84	Subtidal
7	FOR_FOR_LOI	Formigas	Lado oeste do ilhéu	37,270836, -24,781069	WGS84	Subtidal
8	FOR_FOR_LOIba	Formigas	Lado oeste do ilhéu | Destroço de um barco afundado	37,270025, -24,781135	WGS84	Subtidal

**Table 2.** Macroalgae species from Formigas Islets, with information on their relative abundance, origin and status.

**Phylum**	**Species (Accepted Name)**	**Number of records**	**Establishment Means**	**Occurrence Remarks**
Rhodophyta	*Amphiroa beauvoisii* J.V.Lamouroux	1	Native	New record
Rhodophyta	*Asparagopsis armata* Harvey	12	Introduced	New record
Rhodophyta	*Bornetia secundiflora* (J.Agardh) Thuret	4	Native	New record
Rhodophyta	*Botryocladia macaronesica* Afonso-Carrillo, Sobrino, Tittley & Neto	2	Macaronesian endemism	New record
Rhodophyta	*Callithamnion corymbosum* (J.E.Smith) Lyngbye	1	Native	New record
Rhodophyta	*Callithamnion tetragonum* (Withering) S.F.Gray	1	Native	New record
Rhodophyta	*Centroceras clavulatum* (C.Agardh) Montagne	1	Native	New record
Rhodophyta	*Ceramium ciliatum* (J.Ellis) Ducluzeau	2	Native	New record
Rhodophyta	*Ceramium deslongchampsii* Chauvin ex Duby	4	Native	New record
Rhodophyta	*Ceramium diaphanum* (Lightfoot) Roth	4	Native	New record
Rhodophyta	*Ceramium gaditanum* (Clemente) Cremades	8	Uncertain	New record
Rhodophyta	*Chondria capillaris* (Hudson) M.J.Wynne	1	Native	New record
Rhodophyta	*Chondria dasyphylla* (Woodward) C.Agardh	4	Uncertain	New record
Rhodophyta	*Cryptopleura ramosa* (Hudson) L.Newton	12	Native	New record
Rhodophyta	*Dasya ocellata* (Grateloup) Harvey	3	Native	New record
Rhodophyta	*Digenea simplex* (Wulfen) C.Agardh	4	Native	
Rhodophyta	*Dudresnaya verticillata* (Withering) Le Jolis	1	Native	New record
Rhodophyta	*Gelidium spinosum* (S.G.Gmelin) P.C.Silva	1	Native	New record
Rhodophyta	*Gymnogongrus crenulatus* (Turner) J.Agardh	3	Native	New record
Rhodophyta	*Gymnogongrus griffithsiae* (Turner) C.Martius	2	Native	New record
Rhodophyta	*Halurus equisetifolius* (Lightfoot) Kützing	1	Native	
Rhodophyta	*Haraldiophyllum bonnemaisonii* (Kylin) A.D.Zinova	2	Native	New record
Rhodophyta	*Itonoa marginifera* (J.Agardh) Masuda & Guiry	1	Native	New record
Rhodophyta	*Laurencia dendroidea* J.Agardh	6	Introduced	New record
Rhodophyta	*Laurencia viridis* Gil-Rodriguez & Haroun	3	Macaronesian endemism	New record
Rhodophyta	*Leptosiphonia brodiei* (Dillwyn) A.M.Savoie & G.W.Saunders	1	Uncertain	New record
Rhodophyta	*Lophosiphonia obscura* (C.Agardh) Falkenberg	1	Native	New record
Rhodophyta	*Meredithia microphylla* (J.G.Arardh) J.G.Agardh	8	Native	New record
Rhodophyta	*Nemalion elminthoides* (Velley) Batters	6	Native	New record
Rhodophyta	*Neoizziella divaricata* (C.K.Tseng) S.-M.Lin, S.-Y.Yang & Huisman	2	Introduced	New record
Rhodophyta	*Peyssonnelia squamaria* (S.G.Gmelin) Decaisne ex J.Agardh	1	Native	New record
Rhodophyta	*Platoma cyclocolpum* (Montagne) F.Schmitz	4	Native	New record
Rhodophyta	*Plocamium cartilagineum* (Linnaeus) P.S.Dixon	11	Native	New record
Rhodophyta	*Polysiphonia atlantica* Kapraun & J.N.Norris	2	Native	New record
Rhodophyta	*Pterocladiella capillacea* (S.G.Gmelin) Santelices & Hommersand	9	Native	New record
Rhodophyta	*Pyropia leucosticta* (Thuret) Neefus & J.Brodie	1	Native	New record
Rhodophyta	*Rhodymenia holmesii* Ardissone	3	Native	New record
Rhodophyta	*Scinaia furcellata* (Turner) J.Agardh	7	Native	New record
Rhodophyta	*Vertebrata fucoides* (Hudson) Kuntze	1	Uncertain	New record
Chlorophyta	*Bryopsis cupressina* J.V.Lamouroux	2	Native	New record
Chlorophyta	*Bryopsis hypnoides* J.V.Lamouroux	2	Native	New record
Chlorophyta	*Chaetomorpha linum* (O.F.Müller) Kützing	4	Native	New record
Chlorophyta	*Cladophora albida* (Nees) Kützing	2	Native	New record
Chlorophyta	*Cladophora coelothrix* Kützing	1	Native	New record
Chlorophyta	*Cladophora laetevirens* (Dillwyn) Kützing	4	Uncertain	New record
Chlorophyta	*Codium adhaerens* C.Agardh	1	Native	New record
Chlorophyta	*Phyllodictyon anastomosans* (Harvey) Kraft & M.J.Wynne	1	Native	
Chlorophyta	*Ulva compressa* Linnaeus	1	Native	New record
Chlorophyta	*Ulva linza* Linnaeus	2	Native	New record
Chlorophyta	*Ulva rigida* C.Agardh	6	Native	New record
Chlorophyta	*Valonia utricularis* (Roth) C.Agardh	3	Native	New record
Ochrophyta	*Canistrocarpus cervicornis* (Kützing) J.C.De Paula & De Clerck	1	Native	New record
Ochrophyta	*Carpomitra costata* (Stackhouse) Batters	6	Native	
Ochrophyta	*Colpomenia sinuosa* (Mertens ex Roth) Derbès & Solier	7	Native	New record
Ochrophyta	*Cystoseira compressa* (Esper) Gerloff & Nizamuddin	8	Native	New record
Ochrophyta	*Cystoseira humilis* Schousboe ex Kützing	1	Native	New record
Ochrophyta	*Dictyopteris polypodioides* (DC.) J.V.Lamouroux	2	Native	
Ochrophyta	*Dictyota bartayresiana* J.V.Lamouroux	9	Native	New record
Ochrophyta	*Dictyota dichotoma* (Hudson) J.V.Lamouroux	8	Native	New record
Ochrophyta	Dictyota dichotoma var. intricata (C.Agardh) Greville	5	Native	New record
Ochrophyta	*Ectocarpus siliculosus* (Dillwyn) Lyngberg	6	Uncertain	New record
Ochrophyta	*Halopteris filicina* (Grateloup) Kützing	7	Native	
Ochrophyta	*Halopteris scoparia* (Linnaeus) Sauvageau	3	Native	New record
Ochrophyta	*Hydroclathrus tilesii* (Endlicher) Santiañez & M.J.Wynne	1	Introduced	New record
Ochrophyta	*Laminaria ochroleuca* Bachelot de la Pylaie	7	Native	
Ochrophyta	*Padina pavonica* (Linnaeus) Thivy	2	Native	New record
Ochrophyta	*Sargassum cymosum* C.Agardh	4	Native	New record
Ochrophyta	*Sargassum furcatum* Kützing	6	Native	New record
Ochrophyta	*Treptacantha abies-marina* (S.G.Gmelin) Kützing	9	Native	
Ochrophyta	*Zonaria tournefortii* (J.V.Lamouroux) Montagne	13	Native	

**Table 3.** Main taxonomic figures with information on the species origin and status.

**Phyllum**	**Order**	**Family**	**Specimens Number**	**Total taxa**	**Total species**	**Native**	**Intro-duced**	**Uncertain**	**Macaro-nesian endemism**	**New record**
Rhodophyta	13	24	169	54	39	30	3	4	2	37
Chlorophyta	3	6	33	14	12	11		1		11
Ochrophyta	6	7	118	22	19	17	1	1		13
**Total**	**22**	**37**	**320**	**90**	**70**	**58**	**4**	**6**	**2**	**61**
